# Concordance between General Practitioners and Radiation Oncologists for Cancer Follow-Up Care

**DOI:** 10.3390/ijerph20010108

**Published:** 2022-12-21

**Authors:** Tiffany Sandell, Andrew Miller, Heike Schütze

**Affiliations:** 1 Faculty of Science, Medicine and Health, University of Wollongong, Wollongong, NSW 2522, Australia; 2 Radiation Oncology Service, Illawarra Shoalhaven Local Health District, Wollongong, NSW 2500, Australia; 3 Centre for Primary Health Care and Equity, Faculty of Medicine, University of New South Wales, Sydney, NSW 2052, Australia

**Keywords:** cancer, follow-up, general practitioner, shared care, oncologist, concordance, level of agreement

## Abstract

(1) Background: Patients treated with radiotherapy require follow-up care to detect and treat acute and late side effects, and to monitor for recurrence. The increasing demand for follow-up care poses a challenge for specialists and general practitioners. There is a perception that general practitioners do not have the specialised knowledge of treatment side effects and how to manage these. Knowing the concordance between general practitioner and oncologist clinical assessments can improve confidence in healthcare professionals. This study aimed to measure the level of agreement between general practitioners and radiation oncologists using a standardised clinical assessment; (2) Methods: a cross-sectional clinical practice study; sample aim of 20 breast, prostate or colorectal patients, three years post-radiotherapy treatment; their general practitioner and radiation oncologist; (3) Results: There was acceptable percent agreement (>75%) and a moderate to almost perfect agreement (Fleiss kappa) for all variables between the 15 general practitioner-radiation oncologist dyads; (4) Conclusions: The general practitioner and radiation oncologist concordance of a clinical follow-up assessment for radiation oncology patients is an important finding. These results can reassure both general practitioners and oncologists that general practitioners can provide cancer follow-up care. However, further studies are warranted to confirm the findings and improve reassurance for health professionals.

## 1. Introduction

Patients treated with radiotherapy require follow-up care to detect and treat acute, consequential and late side effects specific to radiotherapy, and monitor for recurrence [1]. Acute side effects generally occur during treatment, persist up to a few weeks after treatment, and usually involve intermitotic cells (skin and mucosa). Consequential side effects occur when acute complications persist for longer durations and cause persistent damage [2], whereas late side effects emerge months to years after radiotherapy treatment and usually involve postmitotic cells (liver, kidney, heart, muscle and bone) [1]. The most common follow-up model of care to manage these side effects is the specialist-led model, where the radiation oncologist oversees the care, usually in a hospital setting. However, improved screening, earlier detection and increased incidence of cancer have led to an increased demand for cancer-related services [3] and, subsequently, follow-up care [4]. This increase in the demand for follow-up care poses a challenge to specialists and general practitioners to provide optimal follow-up care [5].

It has been recommended that general practitioners take a greater role in cancer follow-up care [6,7,8,9] in the form of shared care. Shared care is a collaborative process through which different professional groups work together to improve health care quality [10], in this case, the patient’s general practitioner and oncologist. The evidence for the benefits of shared cancer follow-up models of care is growing [11,12,13,14], with randomised controlled trials finding no difference in recurrence or quality of life when a general practitioner is involved in cancer follow-up care compared to an oncologist [15,16,17,18]. Yet, there are still barriers to implementing shared cancer follow-up care into practice.

A systematic review examined the factors that influence the translation of shared cancer care into practice [19] and found that general practitioners were willing to take a greater role in cancer follow-up care, however, general practitioners sought specific follow-up clinical management guidelines to support them, which were based on best practice and preferably written by oncologists. The follow-up assessments could be in the form of a printable checklist or validated instruments and would reassure general practitioners that they are addressing aspects critical for the particular patients’ care [20,21,22,23]. However, some oncologists felt that general practitioners did not have the specialised knowledge of specific treatment side effects and how to manage the side effects [24,25,26], thus posing a barrier in the move to shared cancer follow-up care.

Establishing concordance between health professionals is important as models of care adapt to the ever-changing demands of optimal patient management. However, there is limited research on the concordance (or discordance) of general practitioners and specialists in healthcare. It is important to understand the concordance to improve general practitioner confidence in providing follow-up care and reduce oncologists’ hesitancy to transfer some aspects of care for low-risk patients. Therefore, this study aimed to create a standardised follow-up clinical assessment for general practitioners and radiation oncologists to use on patients previously treated with radiotherapy and measure the level of agreement between general practitioners and radiation oncologists. To our knowledge, this is the first study of its kind.

## 2. Materials and Methods

This was a cross-sectional clinical practice study at the Illawarra Cancer Care Centre (regional) and the Shoalhaven Cancer Care Centre (rural), Australia. The radiation oncology services within these centres were familiar with a web-based health technology and had an existing system that could be integrated into the primary care setting. Ethics approval was received from the Joint University of Wollongong and the Illawarra Shoalhaven Local Health District Human Research Ethics Committee (2020/ETH00301). A detailed protocol is available [27]; the trial was registered with the Australian New Zealand Clinical Trials Registry on 20 October 2020, ACTRN12620001083987.

### 2.1. Sample and Recruitment

Patients were eligible if they were scheduled for a radiation oncology follow-up consultation in 2021 and were three years post-radiotherapy treatment for breast, colorectal or prostate cancer. We purposely selected three years post-radiotherapy as it was expected that patients would have fewer toxicity issues. Radiation oncologists selected patients from their follow-up clinic list that they believed would suit a shared cancer follow-up model of care based on clinical considerations, including treatment type, prescription, and cancer staging.

The sample was taken from the overarching study with a recruitment aim of 20 patients. Patients were sent a participant information sheet informing them about the study aims, processes and inviting them to participate. Once patients consented in writing, their general practitioner was invited to participate.

### 2.2. General Practitioner Training

All general practitioners were visited by a radiation oncologist who provided a demonstration of how to access and complete the follow-up clinical assessment. In addition to this, the research team designed a training program that was approved for continuing professional development points. The training program included information on radiation oncology side effects and management, with a video demonstration on conducting a clinical assessment.

### 2.3. Web-Based Technology

The hospital’s PROsaiq^®^ platform [28] was used to administer clinical assessments between oncologists and general practice via HealthPathways (described further below). PROsaiq consists of a webserver that uses the clinical assessments existing within the Oncology Information System (OIS), to produce a webpage in Xform format with a specific Uniform Resource Locator (URL) that can be shared. The webform contains placeholders for patient identifiers and survey assessment items. When the form is submitted, the webserver alters the returned survey from a JavaScript Object Notation (JSON) format into HL7 format and imports it into the OIS through the usual HL7 gateway. The submitted answers are stored and appear as if the survey had been completed entirely within the OIS. The PROsaiq system also included a module to monitor rejected incoming assessments to allow for manual correction of contained errors, for example, incorrect spelling of the surname, switching of first and last names, or incorrect medical record number. The first author monitored this portal.

The system was trialled for collecting quality of life-based patient-reported outcomes and deemed feasible in terms of use [29,30]. The PROsaiq platform acts only as a server of empty forms and a converter of returned forms, not storing any patient data and deliberately cleaning RAM after the completion of conversion and transfer.

### 2.4. Tool Development

At the time of writing, the cancer centres had an internal Dashboard system using PROsaiq that linked directly to the OIS for follow-up consultations. This allowed for the follow-up process to be streamlined internally. The assessments were compiled internally at the Illawarra and Shoalhaven Cancer Care Centres for follow-up of radiotherapy patients and were based on current practice. These clinical assessments review physical items on a scale from 0 to 3 or 4 (see Table 1) for items specific to radiotherapy follow-up care, such as pain, fatigue, physical performance, bowel issues, urinary issues, and appetite. The included scales were sourced from the Radiation Therapy Oncology Group scales [31] and the Common Terminology Criteria for Adverse Events, version 3.0 [32]. Table 2 displays the items that apply for breast, prostate and colorectal follow-up care.

### 2.5. Data Collection

Radiation oncologist data—

Patients attended their standard scheduled radiation oncology follow-up appointment with their radiation oncologist. The radiation oncologists were provided with a list of patients before their clinic to remind them to enter the data at that point in time, instead of retrospectively. The radiation oncologist entered the clinical assessment data directly into Oncology Information System (OIS: Mosaiq^®^) as part of their standard consultation practice.

General practitioner data—

The patient was requested to visit their general practitioner the day before, the day of, or the day after their radiation oncologist follow-up appointment. This was to try and reflect contemporaneous results for the clinical assessments. The general practitioner accessed the clinical assessment via HealthPathways. HealthPathways is a web-based information portal being implemented in New Zealand, Australia, and the United Kingdom to help general practitioners make assessment, management, and specialist request decisions. Each ‘pathway’ is an agreement between the primary and specialist services, it is developed by specialists or hospital staff and is reviewed and approved by general practitioners.

**Figure 1:**
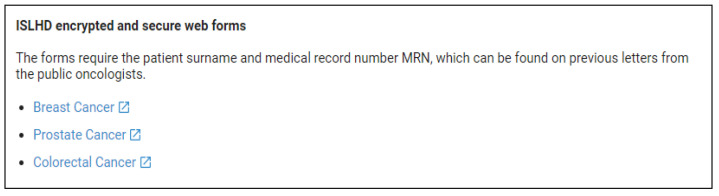
Example of HealthPathways interface and accessing the clinical assessment.

To access the assessment, the general practitioner would first select the relevant cancer type based on the patient (see Figure 1), enter the patient’s name and medical record, and complete the clinical assessment.

### 2.6. Data Analysis

The general practitioner and radiation oncologist data were extracted separately from the OIS, copied and pasted into the same Microsoft Excel spreadsheet for analysis. The data were matched using the patient’s medical record number, and de-identified. Percent agreement and Fleiss’ Kappa was used to determine the level of agreement of the clinical items outlined in Table 1. A 75–90% per cent agreement demonstrates an acceptable level of agreement [33]. Fleiss kappa accounts for chance and is used when the raters are different (four radiation oncologists were the non-unique raters). Fleiss kappa also shows the level of agreement within the variable for each category. Level of agreement is measured from 0 to 1 (slight agreement is <0.20; fair agreement 0.21–0.40; is moderate 0.41–0.60; substantial 0.61–80; and 0.81–1.00 is almost perfect agreement) [34].

## 3. Results

Results were returned on 15 general practitioner-radiation oncologist dyads of data for analysis from the recruited sample of 19 (79%). For participant demographics, see Table 3. Seven dyads were from the Shoalhaven (rural) and eight dyads from the Illawarra (regional); seven were breast cancer patients, seven were prostate patients, and one was a colorectal patient.

The percent agreement between general practitioners and radiation oncologists was above an acceptable level >75% for every variable. The Fleiss kappa ranged from moderate agreement to almost perfect agreement for the variables, see Table 4, and sub analysis by demographic variable was therefore not required.

The clinical items with 100% percent agreement and almost perfect agreement (Fleiss Kappa) were Lymphoedema (all seven patients scored grade 0), recurrence (all seven patients scored grade 0), erectile dysfunction (all seven patients scored grade 3), proctitis and rectal haemorrhage. Fatigue and physical performance had an overall percent level of agreement of 87% and substantial Fleiss Kappa agreement (0.695, 0.0659). General practitioners reported seven patients as experiencing some level of fatigue, and radiation oncologists scored four patients with fatigue. For physical performance, two patients were scored as having some form of physical restriction by the general practitioner, compared to five patients being scored with physical restriction by the radiation oncologist. Appetite and unintended weight change agreement was moderate.

## 4. Discussion

There is limited research in understanding the concordance in knowledge, care patterns, and care outcomes between general practitioners and specialists for many health conditions. This knowledge of concordance (or discordance) is important when evaluating the feasibility of new models of care to manage both the clinical load and patients’ needs and preferences. For example, a study on the concordance of cardiac right bundle branch block diagnosis between cardiologists and general practitioners had substantial agreement, indicating general practitioners had scope to be more involved [35]. However, a study on the diagnosis of skin conditions between general practitioners and dermatologists had moderate agreement, indicating more training was needed if the model was to change [36].

Our study aimed to address two of the barriers to shared cancer follow-up care: the provision of clinical follow-up guidelines; and the perception that general practitioners may lack cancer specific skills and knowledge for shared cancer follow-up care. Our study has demonstrated a moderate to almost perfect agreement between the general practitioner and radiation oncologist completing a prescribed follow-up clinical assessment. The difference in reporting fatigue may be due to the radiation oncologist recording fatigue attributable to radiotherapy treatment from pre-treatment levels, compared to the general practitioner recording general tiredness level. Overall the results indicate general practitioners can adequately assess some aspects of cancer patients during follow-up for radiotherapy side effects and disease recurrence based on a prescribed clinical assessment.

The provision of a follow-up clinical assessment designed by the radiation oncologist is a key component of this research. Studies have suggested the need for specific and localised clinical follow-up guidelines or assessments [20,21]. There are a myriad of programs and software utilised across general practice clinics, and therefore the provision of clinical assessments needs to be easily accessible by general practitioners. Using specific follow-up guidelines that oncologists prepare should reassure both the general practitioner and the radiation oncologist that the general practitioner is addressing critical aspects of the patient’s care [20,23]. Given the moderate to almost perfect agreement we found, these results will be useful to bridge that gap. However, we did not evaluate if using the clinical assessment reassured general practitioners and improved their confidence.

One potential reason that oncologists feel uncertain about general practitioners providing shared follow-up care is that general practitioners rarely contact oncologists unless there is a problem. However, there is an absence in literature regarding general practitioners communicating with oncologists when changes in a patient’s overall condition, co-morbidities or concern for recurrence [37]. Shared cancer follow-up care requires improved communication channels between general practitioners and oncologists in real-time [24]. Our study successfully implemented a system that was easily accessible to general practitioners and allowed them to transfer the consultation results back to the radiation oncologist for review in real-time. Thus the radiation oncologist was not only able to maintain involvement in the patient’s care but could oversee the care. Although there is good concordance between the general practitioner and radiation oncologist, and the radiation oncologist could review the results from the shared care consultation in real-time, we do not know if this has improved the radiation oncologists’ acceptance and the likelihood of engaging in shared care models.

### 4.1. Limitations

This was the first study to our knowledge to evaluate the concordance between general practitioners’ and radiation oncologists’ clinical assessment in cancer follow-up care. A strength of the study was the use of a predefined protocol to minimise subjective clinical review. The limited sample size, which was partly due to the COVID-19 global pandemic and reduced face-to-face consultations, is acknowledged, and the results should be reviewed with caution. In addition, patients were three years post-treatment as it was expected that they would have fewer toxicity issues (as confirmed by our results), and these results cannot be generalised to patients on active treatment or earlier post-treatment. The reporting timeframe may be a factor to consider, as the level of pain or fatigue that a patient reports may be different in the timeframe between appointments of the radiation oncologist and general practitioner.

### 4.2. Future Implications

Future research should consider larger sample sizes, moving the post-treatment follow-up review to earlier in the follow-up phase, and expanding the assessments available for other cancers. Future research should also explore whether the provision of the clinical assessments resulted in improved general practitioner and radiation oncologist reassurance, and confidence that general practitioners can be involved in shared cancer follow-up model of care, and whether good concordance improves acceptance.

## 5. Conclusions

This study supports general practitioners taking a greater role in cancer follow-up care. Collaborative care between general practitioners and oncologists should be further explored. However, it is recognised that there are challenges to translating available evidence into systems to allow health professionals to work both independently and collaboratively to best meet the needs of patients. Systems for shared cancer follow-up care need to be integrated into both health settings and further development and analysis of specific clinical follow-up assessments. Further research with a larger sample, earlier in the post-treatment phase and qualitative analysis is warranted.

## Figures and Tables

**Table 1:** Clinical assessment items and grading scale.

Clinical Assessment Item	Grade
Appetite	0. Normal 1. Decreased, able to eat 2. Hunger, but experiences difficulty with eating 3. Poor, no interest in eating 4. Unable to eat
Chest and breast pain CTCAE 5.0	0. None 1. Mild pain 2. Moderate pain; limiting instrumental activities of daily living (ADL) 3. Severe pain; limiting self care ADL
Diarrhea CTCAE 5.0	0. None 1. Increase of <4 stools per day over baseline; mild increase in ostomy output compared to baseline 2. increase of 4–6 stools per day over baseline; moderate increase in ostomy output compared to baseline; limiting instrumental ADL 3. increase of >=7 stools per day over baseline; hospitalization indicated; severe increase in ostomy output compared to baseline; limiting self care ADL
Dysuria CTCAE 5.0	0. None 1. mild symptoms requiring no intervention 2. symptoms relieved with therapy 3. symptoms not relieved despite therapy
Physical performance ECOG	0. Fully active, able to carry on all pre-disease performance without restriction 1. Restricted in physically strenuous activity but ambulatory and able to carry out work of a light or sedentary nature, e.g., light house work, office work 2. Ambulatory and capable of all selfcare but unable to carry out any work activities; up and about more than 50% of waking hours 3. Capable of only limited selfcare; confined to bed or chair more than 50% of waking hours 4. Completely disabled; cannot carry on any selfcare; totally confined to bed or chair
Erectile dysfunction CTCAE 5.0	0. None 1. Decrease in erectile function, intervention not needed 2. Decrease in erectile function, erectile intervention indicated 3. Decrease in erectile function, erectile intervention not helpful
Fatigue CTCAE 5.0	0. None 1. Fatigue relieved by rest 2. Fatigue not relieved by rest; limiting instrumental ADL 3. Fatigue not relieved by rest, limiting self care ADL
Lymphedema-related fibrosis CTCAE 3.0	0. None 1. minimal to moderate redundant soft tissue that was unresponsive to elevation or compression and that was also firm or spongy 2. Marked increase in density and firmness, with or without tethering 3. Very marked density and firmness with tethering affecting ≥40% of the edematous area
Pelvic pain CTCAE 5.0	0. None 1. Mild pain 2. Moderate pain; limiting instrumental ADL 3. Severe pain; limiting self care ADL
Proctitis CTCAE 5.0	0. None 1. Rectal discomfort, intervention not indicated 2. Symptomatic (e.g., rectal discomfort, passing blood or mucus); medical intervention indicated; limiting instrumental ADL 3. Severe symptoms; fecal urgency or stool incontinence; limiting self care ADL 4. Life-threatening consequences; urgent intervention indicated
Rectal hemorrhage CTCAE 5.0	0. None 1. Mild symptoms; intervention not indicated 2. Moderate symptoms; intervention indicated 3. Transfusion indicated; invasive intervention indicated; hospitalization 4. Life-threatening consequences; urgent intervention indicated
Telangiectasia RTOG	0. None 1. Slight atrophy, pigmentation change, some hair loss 2. Patchy atrophy, moderate telangiectasia, total hair loss 3. Marked atrophy, gross telangiectasia 4. Ulceration
Vomiting CTC	0. None 1. 1 episode in 24 h 2. 2–5 episodes in 24 h 3. ≥6 episodes in 24 h over pretreatment; or need for IV fluids 4. requiring parenteral nutrition; or physiologic consequences requiring intensive care
Weight loss (RTOG)	0. <5.0% 5.0–9.9%, intervention not indicated 1. 10.0–19.9%, nutritional support indicated 2. >=20.0%, tube feeding or TPN indicated

**Table 2:** Radiation oncology follow-up standardised clinical assessment.

Tumour Type	Clinical Assessment
Breast	Fatigue, ECOG (Eastern Cooperative Oncology Group) Performance Status, appetite, weight loss, chest and breast pain, telangiectasia, lymphedema-related fibrosis, and disease state (i.e., local, regional, or distant)
Colorectal	Fatigue, ECOG Performance Status, appetite, weight loss, proctitis, pelvic pain, vomiting, and diarrhea
Prostate	Fatigue, ECOG Performance Status, erectile dysfunction, dysuria, and rectal hemorrhage

**Table 3:** Participant characteristics.

	General Practitioner	Radiation Oncologist	Total
Sex			
Male	9		
Female	6		
Total	15	4 *	19
Age			
30–39	3	1	4
40–49	7	2	9
50–59	2	1	3
60+	3		3
Total	15	4	19
Training			
Australia	6	4	10
Overseas	9		9
Total	15	4	19
Years practising			
<10 years	3	2	5
11–19 years	7	1	8
>20 years	5	1	6
Total	15	4	19
Most recent oncology training			
2–5 years	3	4	7
6–10 years	1		1
>10 years	7		7
No training	4		4
Total	15		19

* Due to the potential to identify participants’ sex has not been itemised.

**Table 4:** Results of level of agreement between general practitioners and radiation oncologists.

Tumour	Variable	N	Percent Agreement	Fleiss Multi Rater Kappa	Significance
All	fatigue	15	87%	0.695	0.003
	ECOG	15	87%	0.659	0.011
Breast and colorectal	Appetite	8	88%	0.590	0.095
	Unintended weight change	8	88%	0.590	0.095
Breast	Chest pain	7	100%	1.00	0.008
	Telangiectasia	7	86%	0.712	0.011
	Lymphoedema	7	100%	1.00	0.008
	Recurrence (local, regional, distant)	7	100%	1.00	0.008
Prostate	Erectile dysfunction	7	100%	1.00	0.008
	Proctitis	7	100%	1.00	0.008
	Rectal	7	100%	1.00	0.008
Prostate and colorectal	Dysuria	8	88%	0.590	0.095
Colorectal	Pelvic pain	1	100%	-	-
	Vomiting	1	100%	-	-
	Diarrhea	1	100%	-	-
	Proctitis	1	100%	-	-

## Data Availability

The dataset generated during and/or analysed during the current study is available from the corresponding author upon reasonable request.
